# Hyperprogression After Immunotherapy for Primary Small Cell Neuroendocrine Carcinoma of the Ureter: A Case Report

**DOI:** 10.3389/fonc.2021.696422

**Published:** 2021-08-17

**Authors:** Defeng Qing, Luxing Peng, Feng Cen, Xinjun Huang, Qiang Wei, Heming Lu

**Affiliations:** ^1^Department of Radiation Oncology, People’s Hospital of Guangxi Zhuang Autonomous Region, Nanning, China; ^2^Department of Pathology, People’s Hospital of Guangxi Zhuang Autonomous Region, Nanning, China

**Keywords:** ureter, small cell neuroendocrine carcinoma, PD-L1, chemoradiotherapy, case report

## Abstract

**Background:**

Primary small cell neuroendocrine carcinoma (SCNEC) in the ureter is extremely rare and has been sporadically reported in case reports. Its incidence, diagnosis, treatment, and outcomes have not yet been thoroughly understood. Here we present a patient with advanced SCNEC in the ureter who was treated by multimodal strategies. To the best of our knowledge, this is the first literature report about the clinical outcomes of the combination of programmed death ligand 1 (PD-L1) immune checkpoint inhibitors (ICIs) and radiotherapy in patient with primary ureteral SCNEC.

**Case Presentation:**

A 71-year old male presented with right flank pain and gross hematuria. A laparoscopic right nephroureterectomy was performed. He was diagnosed with primary ureteral SCNEC, pT3N0M0. Following the surgery, 4 cycles of adjuvant chemotherapy with carboplatin and etoposide (CE) were administered, with disease-free survival (DFS) of 10.1 months. He was then offered 4 cycles of palliative first-line chemotherapy with nedaplatin and irinotecan. The disease was continuously progressed, with progression-free survival (PFS) of 3.7 months. The patient subsequently received second-line treatment with PD-L1 ICI combined with radiotherapy. Unfortunately, hyperprogressive disease was found at the end of treatment. MRI and CT scan showed bilateral pubic bones, right acetabulum, and liver metastases. Without further intervention, the patient died from extensive metastatic disease 2 months after diagnosis, with overall survival (OS) of 18.2 months.

**Conclusion:**

Physicians must be aware of this rare and aggressive carcinoma at its initial presentation. Special attention should be paid to the potential likelihood of hyperprogression during the treatment.

## Highlights

• We present a case with rare disease. • We are the first to report the clinical result of combination of PD-L1 ICIs and radiotherapy in primary ureteral SCNEC. • We observed an unusual side effect in patient treated with ICIs. • The OS of this patient was 18.2 months, superior to those from previous case reports.

## Introduction

Primary small cell neuroendocrine carcinoma (SCNEC) often occurs in the respiratory system. As an extremely rare disease in the urinary tract, it accounts for less than 0.5% of urinary tract malignancies (1, 2). The bladder and prostate are the major sites affected by SCNEC in the urinary tract, while its localization in the ureter is extraordinarily rare, with no more than 50 cases reported in the English literature (3, 4). The biological behavior of this tumor is aggressive. Most primary ureteral SCNECs tend to progress rapidly and its prognosis is dismal, with most deaths occurring within 1 year (4, 5).

The management of ureteral SCNEC depends on the stage of disease at initial diagnosis. The multiple treatment modalities for limited-stage disease contain radical cystectomy, chemotherapy and radiotherapy. However, there is no recommendation for extensive or advanced disease. To the best of our knowledge, there is no related report focusing on PD-L1 ICIs combined with radiotherapy in primary advanced ureteral SCNEC so far. This presentation is the first report about PD-L1 ICI combined with radiotherapy for ureteral SCNEC.

## Case Report

**Figure 1:**
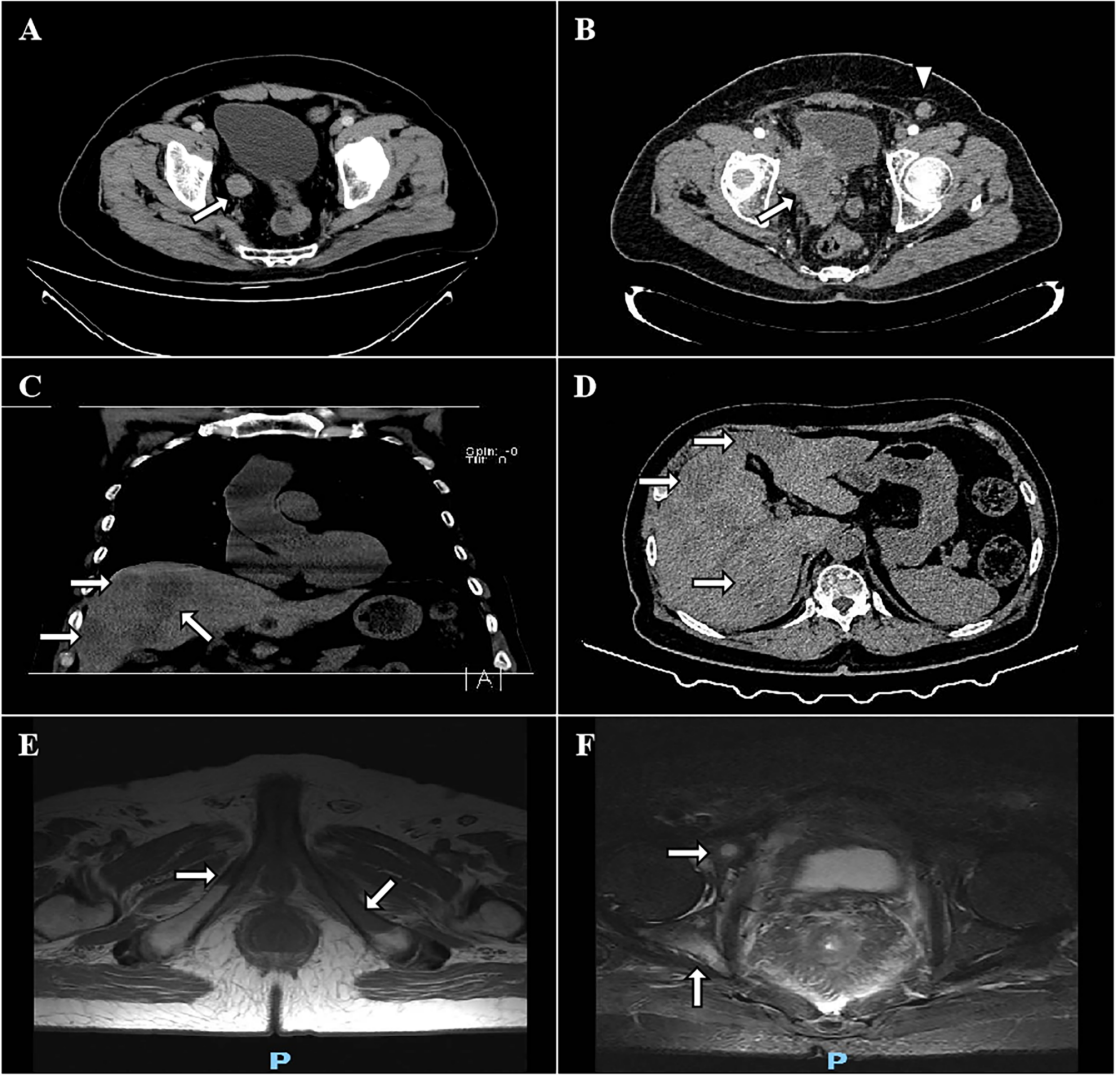
CT and MRI images of the mass. The preoperative transverse view reveals that the tumor was located in the lower segment of the right ureter **(A)**. CT scan reveals local relapse in the tumor bed with infiltration to the surrounding tissues (white arrow, **B**). Left inguinal lymphadenopathy (2.1x1.5 cm, white triangle, **B**). Coronal and transverse views show a progressive SCNEC with multiple liver **(C, D)**, bilateral pubic bones **(E)**, and right acetabulum **(F)** metastases after 2 cycles of durvalumab.

**Figure 2:**
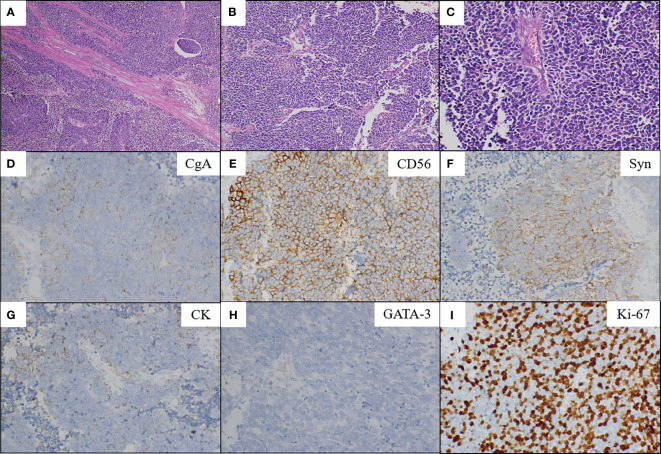
Microscopic findings and immunohistochemical staining. Low (**A**; magnification, x100) and high (**B**; x200, C; x400) power showed the SCNEC (hematoxylin and eosin staining). The tumor is composed of small round cells which had enormous hyperchromatic nuclei and limited cytoplasm with a high nuclear/cytoplasmic ratio **(C)**. Positive immunohistochemical staining for chromogranin-A **(D)**, CD56 **(E)**, synaptophysin **(F)** and CK **(G)**. Negative immunohistochemical staining for GATA-3 **(H)**. The Ki-67 index was 80% **(I)**. CgA, chromogranin-A; Syn, synaptophysin; CK, cytokeratin; GATA-3, GATA binding protein 3.

A 71-year old male presented with right flank pain and gross hematuria to our institution. Physical examination showed pain at the right costovertebral angle, extending to the right groin over the location of the ureter. The performance status of Eastern Cooperative Oncology Group (ECOG) was 1. Abdominal and pelvic contrast-enhanced computed tomography (CT) scan revealed a large mass, measuring about 5 cm in the distal right ureter with moderate right hydroureterosis (**Figure 1A**). Chest CT scan, head magnetic resonance imaging (MRI), and bone emission computed tomography (ECT) showed no evidence of metastases. The clinical stage was cT3N0M0. Then he underwent laparoscopic right nephroureterectomy. Microscopic examination revealed that the tumor was composed of small round cells which had enormous hyperchromatic nuclei and limited cytoplasm with a high nuclear/cytoplasmic ratio (**Figures 2A–C**). The ureteral wall, perineural spaces, and periureteric adipose tissue were infiltrated by these small round tumor cells. The lymphatic vessels invasion were positive. The right external iliac nodes and ureteral resection margins were negative. Upon immunohistochemical test, the neuroendocrine markers (chromogranin-A, synaptophysin, and CD56), and epithelial markers (CK) were positive in the small round tumor cells (**Figures 2D–G**), while negative for urothelial markers (GATA-3) (**Figure 2H**). The mitotic count was high, and the proliferative index counted by Ki-67 was 80% (**Figure 2I**). The final diagnosis was made as SCNEC. The pathological stage at the diagnosis was pT3N0M0. The patient received 4 cycles of CE regimen (Carboplatin at AUC=4 mg/ml.min was infused on day 1, etoposide at 100 mg/m^2^ was infused on days 1-3, every 21 days). The patient had grade 1 gastrointestinal toxicity during periods of adjuvant chemotherapy.

The disease progressed at 7.5 months after the completion of chemotherapy, with DFS of 10.1 months. Pelvic CT scan showed an 8.2x6.8 cm mass infiltrating bladder wall without clear margins to the surrounding tissues (**Figure 1B**). A left inguinal lymphadenopathy measured 2.1x1.5 cm was suspected with metastasis (**Figure 1B**). The patient underwent percutaneous biopsy of this lymph node which was finally confirmed metastatic SCNEC. Palliative first-line chemotherapy was administered with nedaplatin at a dose of 80 mg/m^2^ on day 1 and with irinotecan at a dose of 65 mg/m^2^ on days 1 and 8, every 21 days for 4 courses. The patient experienced grade 3 thrombocytopenia, grade 3 neutropenia and grade 2 diarrhea after the first cycle of chemotherapy, according to the Common Terminology Criteria for Adverse Events (CTCAE) version 4.03, and recover from symptomatic treatment, causing subsequent chemotherapy delayed for 1 week. However, the disease still progressively deteriorated, with PFS of 3.7 months. Chest and cranial CT scan showed no evidence of any other metastases. The symptoms of right thigh numbness, swelling, pain and gross hematuria were emerged again. His ECOG performance status rose to 3. But he was capable of parts of selfcare, had adequate cardiopulmonary, liver and renal functions, and didn’t require oxygen therapy. The patient finally received intensity-modulated radiation therapy (IMRT) concomitantly with 2 cycles of PD-L1 ICI (durvalumab). The prescribed radiation dose was 60 Gy at 2 Gy per fraction over 6 weeks, delivered to the pelvic metastatic mass. Durvalumab at 10 mg/kg was infused on day 1, every 14 days. The patient had not any other clinical signs and symptoms during the combination of radiation and immunotherapy. At the end of radiotherapy, the symptoms of thigh numbness, pain and gross hematuria disappeared. However, MRI and CT scan showed bilateral pubic bones (**Figure 1E**), right acetabulum (**Figure 1F**), and liver metastases (**Figures 1C, D**). The pelvic metastatic disease was stable. Given the deterioration of this patient, he was transferred to a hospice service and died 2 months after diagnosis, with OS of 18.2 months.

## Discussion

Urinary tract extrapulmonary SCNEC most commonly occurs in the bladder and prostate. Primary SCNEC of the ureter is extremely rare, with less than 50 cases reported since it was first described by Ordonez et al. in 1986. Due to its rarity, little is known about the clinicopathologic characteristics and outcomes in these patients. Zhang et al. (6) showed that the natural course and biological features of ureteral SCNEC are different from those of urothelial carcinoma, so the treatment strategies between the two kinds of tumor should be different accordingly.

Primary ureteral SCNEC often occurs in elderly male population. The common manifestations of these tumor are gross hematuria and flank pain (7). As SCNEC originates from pluripotent stem cells with neuroendocrine differentiation, some patients could present with paraneoplastic syndrome (3, 8–10). When these symptoms emerge, it indicates that the disease might be already at extensive or advanced stages. Since SCNEC belongs to a subgroup of oat cell carcinoma with highly aggressive local invasion and distant metastases, the prognosis is poor. Even patients with early stages, the prognosis is still dismal. The median OS is 17 months, with 1- and 3-year OS rates of 51.9% and 30.3%, respectively (6). Currently, standard treatment strategies for ureteral SCNEC have not been well established. Treatment decision-making is mainly based on sporadic case reports and knowledge of small cell lung cancer (SCLC). Kouba et al. (11) considered that primary urinary tract SCNEC has the same clinical characteristics and homologous genetic as the pulmonary small cell cancer (SCC), so the clinical strategies derived from SCLC may provide a novel insight into the treatment of the ureteral SCNEC.

In general, the treatment strategies are based on the stage of disease at initial diagnosis and patient’s performance status. For early-stage disease, radical resection is the primary treatment modality. Surgical approach including radical nephroureterectomy with excision of the bladder cuff is recommended to patients with upper tract urothelial carcinomas (12). Nonetheless, the prognosis is still dismal with high systemic relapse rates. Zhong et al. (6) reported that 3 (9.4%) patients had regional recurrences and 8 (25%) patients experienced distant metastases during a relatively short follow-up time. Ping et al. (9) reported a patient with pT2N0M0 disease had recurrence in the retroperitoneum at 4 months after radical resection. Wang et al. (13) presented a case with stage of pT3N0M0 who had extensive metastases after nephroureterectomy, with OS of 12 months. Previous study (14) revealed that the incidence of recurrence was approximately 60% due to the occult and micro-metastasis at initial diagnosis, indicating that multimodality treatments are mandatory for ureteral SCNEC.

Theoretically, ureteral SCNEC could benefit from chemoradiotherapy as the pulmonary SCC, which has been demonstrated by plenty of researches. Recommendations for limited-stage SCLC is concurrent chemoradiotherapy rather than cystectomy. According to experiences from SCLC, radiotherapy can replace cystectomy as local consolidative treatment, resulting in a long-term disease control. Regarding limited-stage ureteral SCNEC, however, whether concurrent chemoradiotherapy can replace resection needs to be further evaluated.

Platinum-based doublet chemotherapy regimen was recommended in SCC. EP (etoposide and cisplatin) or CE (carboplatin and etoposide) regimen is one of the classic front-line therapies for this tumor, with a response rate of 69% (15). Adjuvant chemotherapy could reduce the risk of early disease progression compared to surgery alone. Previous researches indicated that surgery alone was not optimal, with a median OS of 8.2 months, and surgery combined with chemotherapy could resulted in favorable long-term survival outcomes (5). Ouzzane et al. (7) showed that the median OS for adjuvant chemotherapy and surgery alone were 24 and 12 months (p=0.56), respectively. In the present case, in order to reduce the risk of complications associated with surgery, the patient received 4 cycles of CE regimen with DFS of 10.1 months. While there is no standard further treatments for ureteral SCNEC so far. Systemic salvage chemotherapy regimens in metastatic ureteral SCNEC are sometimes chosen based on experience in SCNEC originating from other organ sites. The patient then received 4 cycles of palliative first-line chemotherapy with irinotecan plus nedaplatin. However, the disease continuously progressed with PFS of 3.7 months. In addition, grade 3 acute toxicities should not be ignored. The clinical results revealed that ureteral SCNEC was not as sensitive to chemotherapy as other SCC.

In the past decade, due to the fact of the introduction of whole-body radiation to ablate the patient’s immune system in preparation for allogeneic transplant, radiotherapy is typically regarded as an immunosuppressive modality (16). Ionizing radiation activates potential transforming growth factor β cytokine, promotes accumulation of Treg cells and enhances the immunosuppressive effect of M2 macrophages (17–19). Nowadays, effects of local radiotherapy in metastatic disease are rapidly emerging as opportunities to remodel and enhance immunity against cancer, with proimmunogenic effects prevailing over immunosuppressive effects. Radiation promotes the priming and effector phases of the antitumor immune response by lots of cytokines and chemokines, such as CRT, HMGB1, IL-1β, IFN-γ, CXCL9, CXCL10, CXCL16, CD80,VCAM-1, ICAM-1, NKG2DL and so on (20). Plenty of preclinical data have supplied sufficient evidence to propose that radiation is recognized as contributing to systemic antitumor immunity. They hold that the role of palliative radiotherapy in metastatic disease is changing into that of a powerful adjuvant for immunotherapy. The results from radiotherapy in treating ureteral SCNEC are extremely rare. For patients with SCC of the urinary bladder, radiotherapy alone is typically applied to metastatic diseases, such as painful bone or brain metastases (21). Beddok et al. (22) presented a case with common iliac lymphadenopathy metastasis treated with radiotherapy at a prescribed dose of 46.8 Gy at 1.8 Gy per fraction, with complete response of 16 months. Jang et al. (23) showed that surgery followed by radiotherapy at a dose of 54 Gy at 1.8 Gy per fraction to the tumor bed was well tolerated, feasible and effective, with DFS of 10 months. Given the rarity of ureteral SCNEC, currently, there is no recommendations for either radiotherapy technique, target definition, total dose, or dose fraction. The influence of prophylactic cranial irradiation (PCI) on SCLC has been generally acknowledged by lots of investigators, whether patients with ureteral SCNEC can benefit from PCI needs to be further evaluated.

The addition of PD-L1 ICIs in treating patients with SCLC has been recently demonstrated to be safe and effective in a meta-analysis of randomized clinical trials (24). Despite no records on the efficacy of ICIs in ureteral SCNEC, we followed SCLC treatment guidelines and preferred durvalumab over second-line chemotherapy due to the fact of the poor efficacy of chemotherapy and the absence of strong recommendations for further treatments in ureteral SCNEC. We are the first to report this combination of radiation and immunotherapy for this tumor. In the present case, the patient received durvalumab with concurrent IMRT for 2 cycles. Despite the absence of unpleasant symptoms, metastases to the bilateral pubic bones, right acetabulum and liver were found at the end of IMRT. The clinical results indicated that the combination of durvalumab had a weak effect on PFS, consistent with previous researches. The phase III CASPIAN study revealed that the combination of durvalumab significantly improved overall response rate (ORR) and prolonged OS in extensive-stage SCLC, whereas PFS could not benefit from it (5.1 versus 5.4 months) (25). Updated findings presented at the 2020 ESMO annual meeting (26) showed that patients with SCLC treated with durvalumab had a similar proportion (8.3% versus 9.5%) developed brain metastases compared to those without it.

Along with the increasing use of ICIs, more and more different kind of side effects, such as hyperprogression, have been reported. Hyperprogression refers to tumor in primary or metastatic site which experiences a sharp paradoxical progression in a quite short periods. Hyperprogression is defined as disease progression acceleration with tumor burden exceeding 50%, more than 2-fold increase of the tumor growth rate, or time-to-treatment failure less than 8 weeks (27, 28). Champiat et al. found hyperprogressive disease have no relationship with the tumor burden at baseline, the number of metastatic sites, poor performance status, the number of previous lines, and the type of previous treatment line. Multivariate linear regression analysis showed that only the tumor growth rate and age were associated with hyperprogression (28). The mechanism of hyperprogression is not unequivocal. Some previous hypothesis considered that the combination of radiotherapy might be a potential risk factor in the development of hyperprogression. Radiation could produce plenty of tumor antigens which modulate the immune microenvironment that facilitate disease accelerated progression. In the present case, the ICI was administered as a treatment option in the second-line setting, and this is the moment where disease begins to rapid progress and performance begins to deteriorate as well. Whether it is the adverse effect of ICI or the natural course of the disease is largely unknown. Still, these new combination therapeutic viewpoint bring about some questions. The first one is the potentially increased risk of high-grade toxicity caused by combination treatment. Second, in terms of the optimal regimens of radiotherapy, whether conventional fractionated radiotherapy or stereotactic body radiotherapy when combined with ICIs is controversial. And the most important is the optimal therapeutic sequence for advantageous combination with available ICIs. In addition, new molecular therapeutics are required to improve prognosis for this rare and lethal carcinoma. Agents targeting EGFR, C-kit, BCL-2, CD56, and platelet-derived growth factor receptor-α might be promising therapeutic approaches (29, 30).

## Conclusion

Primary ureteral SCNEC is an extremely rare, insensitive, and aggressive disease, with a poor prognosis. We are the first to report a case of primary ureteral SCNEC treated with multimodality strategies, particularly focusing on the clinical result of the combination of PD-L1 ICIs and radiotherapy. Although the outcome was far from satisfactory, it did provide a novel insight into the treatment of the primary ureteral SCNEC. Additionally, the possibility of hyperprogression during immunotherapy should be taken into account.

## Data Availability Statement

The original contributions presented in the study are included in the article/supplementary material. Further inquiries can be directed to the corresponding author.

## Ethics Statement

The studies involving human participants were reviewed and approved by People’s Hospital of Guangxi Zhuang Autonomous Region. The patients/participants provided their written informed consent to participate in this study. Written informed consent was obtained from the individual(s) for the publication of any potentially identifiable images or data included in this article.

## Author Contributions

Conceptualization: DQ and HL. Writing – original draft: DQ and LP. Writing – editing: HL. Data curation: DQ, LP, FC, XH, and QW. Formal analysis: DQ and LP. Supervision: HL. All authors contributed to the article and approved the submitted version.

## Conflict of Interest

The authors declare that the research was conducted in the absence of any commercial or financial relationships that could be construed as a potential conflict of interest.

## Publisher’s Note

All claims expressed in this article are solely those of the authors and do not necessarily represent those of their affiliated organizations, or those of the publisher, the editors and the reviewers. Any product that may be evaluated in this article, or claim that may be made by its manufacturer, is not guaranteed or endorsed by the publisher.
